# Psychosocial impact of prognostic genetic testing in uveal melanoma patients: a controlled prospective clinical observational study

**DOI:** 10.1186/s40359-020-0371-3

**Published:** 2020-01-31

**Authors:** Marietta Lieb, Sefik Tagay, Anja Breidenstein, Tobias Hepp, Claudia H. D. Le Guin, Jennifer Scheel, Dietmar R. Lohmann, Norbert Bornfeld, Martin Teufel, Yesim Erim

**Affiliations:** 1 Department of Psychosomatic Medicine and Psychotherapy, Friedrich-Alexander-University Erlangen-Nürnberg (FAU), Erlangen, Germany; 2 Department of Psychosomatic Medicine and Psychotherapy, LVR Hospital Essen, University of Duisburg-Essen, Essen, Germany; 3 Department of Applied Social Sciences, University of Technology, Cologne, Germany; 4 Institute of Medical Informatics, Biometry and Epidemiology, Friedrich-Alexander-University Erlangen-Nürnberg (FAU), Erlangen, Germany; 5 Department of Ophthalmology, University Hospital Essen, University Duisburg-Essen, Essen, Germany; 6 Center for Health Services Research in Medicine, Department of Psychiatry and Psychotherapy, University Hospital Erlangen, Friedrich- Alexander-University Erlangen-Nürnberg (FAU), Erlangen, Germany; 7 Institute of Human Genetics, University Hospital Essen, University Duisburg-Essen, Essen, Germany

**Keywords:** Prognostic genetic testing, Uveal melanoma, Psychosocial impact, Controlled prospective clinical observation trial, Psycho-oncological intervention

## Abstract

**Background:**

The risk of metastases in uveal melanoma can accurately be estimated through genetic analysis of the tumor. A growing number of patients decide to receive information on their prognosis, although this can be extremely burdensome. Studies on the psychosocial impact of testing are sparse. The objective of this study was to examine traits of patients opting for prognostication, to investigate its psychosocial impact and the use of psycho-oncological services over time. We further examined characteristics of patients utilizing these services and risk factors of prolonged psychological distress.

**Design and methods:**

This study is a non-randomized controlled prospective clinical observational trial. Patients availing for prognostication formed the test group, while those who opted out constituted the observational group. The psychosocial impact of genetic testing was assessed with the following variables: resilience, social support, fear of tumor progression, depression, general distress, health-related quality of life, estimation of the perceived risk, and the utilization of psycho-oncological interventions. Data were assessed at five different time points over a period of 12 months. We applied binary logistic regression analysis, multiple linear regressions and a mixed model.

**Results:**

Of 175 patients, 63 decided to obtain prognostic information. Treatment method (enucleation > brachytherapy), lower social support and higher general distress could significantly predict patient’s choice for prognostic testing. After result announcement, perceived risk of metastases was significantly increased in patients with poor prognosis, while it decreased in those with good prognosis. Overall, a significant decrease over time appeared concerning fear of progression, general distress, depression and anxiety. Mental quality of life increased over time. The utilization of psycho-oncological interventions increased significantly after prognostication; however, this was equivalent in the test and observational groups. Female sex, higher general distress and higher anxiety predicted greater use of psycho-oncological interventions.

**Discussion:**

Availing of prognostic testing is not associated with poorer subsequent psychological well-being. It rather may help to alleviate distress and promote a more realistic risk perception. However, psychological support should be available to all patients, independent of prognosis and treatment, especially considering that patients with low social support and high distress increasingly opt for prognostication.

## Background

Uveal melanoma (UM) is the most common primary intraocular tumor in adult patients with incidence rates of 1.3–8.6 per million in Europe [1]. Novel techniques in tumor genetics profiling yield the possibility of classifying UM in two different biological classes [2]. Metastasis risk and thus mortality rates vary considerably between tumor types [3, 4]. For tumors classified as Monosomy 3 (M3), a ten-year disease-specific mortality of 55% was found, while it dropped to 0% for Disomy 3 (D3) [4]. In a study of enucleated patients, death rates of 75.1% could be observed for M3, whereas D3 was associated with a distinctly lower rate of 13.2% (median follow-up time 5.2 years) [5]. Since effective therapies for patients with metastases are lacking, prognostic testing does not affect treatment decisions nor does it benefit survival [6, 7]. Yet a growing number of patients with UM request to receive information on their prognosis [8, 9]. Primary reasons for this decision are better life-planning, a higher sense of control and hopefulness [8–10]. Nonetheless, prognostication is likely to be emotionally burdensome due to its dichotomous outcome (D3 = good prognosis, M3 = poor prognosis).

Studies investigating the psychosocial impact of prognostic genetic testing in UM patients are sparse and yield inconsistent results: In a retrospective study [9] depressive symptoms as well as mental and physical quality of life were found to be independent of the test result and equal to an age-matched population sample. Another research group [8] similarly found no evidence of patients being harmed or experiencing trouble coping with the information. They concluded that since patients (even with poor prognoses) did not express any regrets about their decision, testing did not yield adverse consequences. In a recent prospective study [11], depression, anxiety and decision regret were examined prior to, 3 and 12 months after testing in UM patients. Independent of their prognosis, patients showed higher depression and anxiety scores at baseline with a decline over time. Decision regret peaked at result announcement, but also diminished afterwards. Hope-Stone and colleagues [12] examined UM patients’ quality of life, anxiety, and depression scores at different time points after treatment. Patients with a confirmed diagnosis of M3 continuously showed higher depression, although the mean score did not reach the cut-off for clinical depression. A comparable result was encountered by Reimer and colleagues [13]. They found heightened distress and a reduced quality of life in patients diagnosed with malignant uveal melanoma (M3) compared to the healthy norm and other ophthalmological patients. There was even a further decrease post-treatment (radiotherapy), implying a possible influence of treatment [13]. Some studies suggest a deterioration of global or vision specific quality of life and mental health depending on treatment modality (Enucleation vs. Brachytherapy) [14–19]. For instance, enucleation can result in some degree of visual loss, cosmetic changes, body image problems, or avoidance of social events [12, 17, 20]. Other studies, however, could not find relevant differences [10, 12, 20, 21]. Overall, research on effects of treatment and genetic testing remains heterogeneous.

Moreover, it appears that psychological impact of genetic testing is not only the result of the communicated cancer risk, but can be mediated by the patients’ interpretation and perception of this risk [22]. Since probabilities are rather understood intuitively, prognostication most likely does not reduce uncertainty for any prognosis in UM (both M3 and D3) [23]. Furthermore, poor prognoses were even interpreted as hopeful. Closer medical contact and thus a higher chance of detecting metastases were considered to maximize survival chances, despite having received the information that screening does not prolong life [8, 24].

Another important issue besides the question of why patients undergo genetic testing is which individuals decide to receive prognostic information. Beran and colleagues [9] investigated sociodemographic factors in UM patients and found that male sex, lower income, and a more recent treatment increased the likelihood of opting for prognostic testing. However, most UM patients do not seem to make a rational or active decision and simply trust in what the clinicians offer them [24]. Some do not even recall being offered either the test or the result, probably due to the emotionality of the situation [9, 24]. In general, patients seem to prefer a shared or passive role in the decision making process and only few of them strive for autonomy [25]. Moreover, UM patients show a large variety of unmet supportive care needs [26] and a wish for psychological counseling both before and after result announcement [9]. Therefore, offering psycho-oncological intervention is considered vital [10, 27]. Overall, there still seems to be a lack of consensus in the area of genetic testing of UM patients [10]. Which patients are opting for prognostic testing and how those patients are impacted by diagnostic results is largely unexplored. Further research is necessary to clarify which patients increasingly utilize psycho-oncological interventions and who is especially at risk for adverse psychological effects. To our knowledge, no prospective controlled study exists in this field. By overcoming shortcomings of previous research like retrospective [9] and uncontrolled [11, 12] investigations, as well as qualitative approaches with small sample sizes [8, 23, 24], we wish to shed more light on this topic.

## Design and methods

### Objectives of the study

The primary aim of this study was to investigate the psychosocial impact of prognostic genetic testing in patients with uveal melanoma. The following three issues were addressed: 1) Decision-making: Which characteristics do patients display who opt for genetic testing? 2) Psychological Distress: How distressing is genetic testing and how do psychological parameters change after the results of tests are revealed? Which patients utilize psycho-oncological interventions and how does the use of these interventions change over time? 3) Risk: Which factors predict prolonged psychological distress? This study focuses on the potentially different trajectories and characteristics of patients deciding for or against prognostic genetic testing.

### Study population and recruitment methods

Inclusion criteria for the study were the diagnosis of uveal melanoma and the possibility of tumor sample removal (enucleation, biopsy or transretinal endoresection) if genetic testing was requested by the patient. Participants had to be at least 18 years old, have sufficient knowledge of the German language and give informed consent to participate in the study. Patients with a pre-existing diagnosis of mental disability, psychosis or dementia were excluded. The sample was consecutively derived from patients undergoing cancer therapy at the Department of Ophthalmology of the University Hospital in Essen.

### Study design and allocation to groups

This non-randomized controlled prospective clinical observational trial is the result of three collaborating networks (Department of Psychosomatic Medicine and Psychotherapy of the University Hospital in Erlangen and Essen, Department of Ophthalmology of the University Hospital Essen, Department of Human Genetics of the University Hospital Essen), funded by the German Cancer aid (authorization number: 110961). Allocation to groups was done by patients’ choice (non-randomized). Patients who availed of prognostic testing were allocated to the Test Group, while those who opted out formed the Observational Group. Blinding was not feasible.

### Measurement points and instruments

**Fig. 1:**
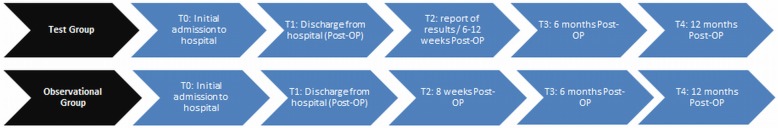
Timeline of study procedure for both groups

All patients were assessed at five different time points as depicted in Fig. 1.

At baseline assessment (t0), sociodemographic data, medical history, and initial protective factors (resilience and perceived social support) were collected. Repeated measures (t0-t4) were carried out for psychological distress (general distress, fear of progression, depression), health-related quality of life, and estimation of perceived risk of metastases. At t0, patients were informed that utilization of psycho-oncological interventions is possible. These interventions took place as inpatient or outpatient treatment with an approximate duration of 50 min each and were conducted by a clinical psychologist specialized in psycho-oncology. The interventions contained elements of resource activation, relaxation techniques (breathing exercises and imaginations), and containment of disease-related affects.

Frequency of use and satisfaction were registered (t1-t4). The variables assessed at specific time points including measurement methods are displayed in Table 1.

**Table 1:** Measurement methods and time points

Psychological Variables	Measurement Method	Description	t0	t1	t2	t3	t4
Resilience	SOC-13 [28]	13 Items, range: 7–91	√	–	–	–	–
Social Support	SozU-K-22 [29–31]	22 Items, range: 1–5	√	–	–	–	–
Fear of Progression	Fear of Progression Questionnaire [32, 33]	43 Items, range: 1–5	√	√	√	√	√
General Distress	Distress Thermometer [34]	1 Item. range: 0–10	√	√	√	√	√
Depression and Anxiety	HADS-D [35]	14 Items; Depression range: 0–21; Anxiety range: 0–21	√	√	√	√	√
Health-related quality of life (Mental and Physical)	SF-12 [36]	12 items, Mental range: 12.60–71.80, Physical range: 11.67–64.92	√	√	√	√	√
Perceived Risk	Visual Analogue Scale (VAS)	1 Item, range: 0–10	√	√	√	√	√
Utilization of and satisfaction with psycho-oncological interventions	Documentation form	Qualitative: Frequency, satisfaction	–	√	√	√	√

### Statistical analysis

For descriptive statistics, we depicted mean values and standard deviations. We used Independent T-tests, Mann-Whitney U-tests, and Chi-square tests to examine potential differences in characteristics between patients of the Test Group and the Observational Group. A binary logistic regression was conducted to examine factors predicting the decision for or against genetic testing. Moreover, mixed models were used to examine the course of psychological variables over time with focus on changes in the Test Group after diagnosis and on the comparison to the Observational Group while controlling for age, sex and enucleation. We controlled for enucleation since loss of sight and changes in appearance can entail diverse impairments, for example deterioration of eye-related quality of life [17, 18]. To analyze which psychosocial variables predict prolonged psychological distress (Depression, Anxiety, and General Distress at t4), linear regression analyses were conducted. Overall, approximately 1% of items were missing from the dataset. EM-Imputations were used to replace single missing values per psychometric scale. Missing values for whole measurement points (e.g. due to dropouts) were not replaced. All results were interpreted on a significance level of *p* < .05. Data were processed using the software SPSS 21 for Microsoft Windows©. Estimation of mixed models was carried out using the nlme-package [37] and the lme4-package [38] for R version 3.5.1 [39].

## Results

**Fig. 2:**
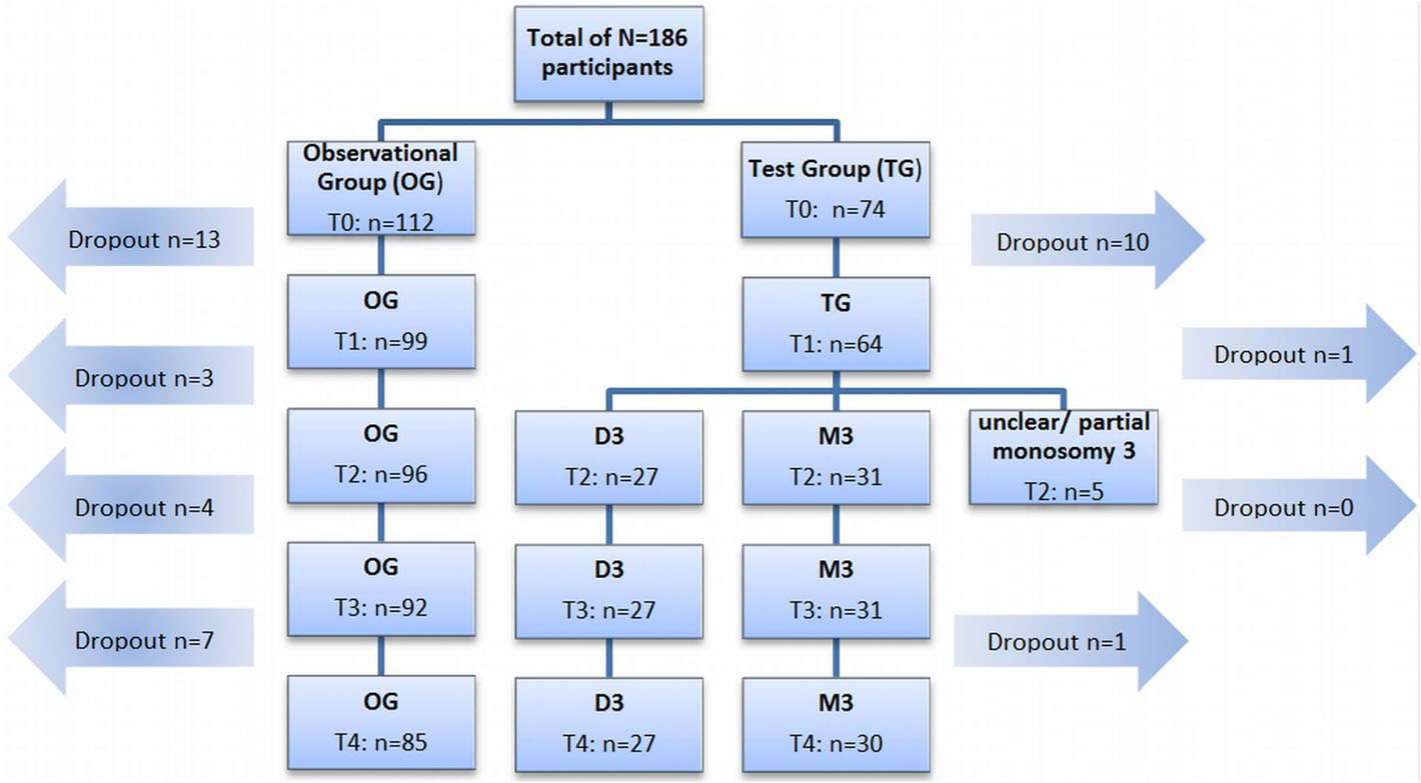
Flow-Chart for group allocation and drop-outs. D3 = Disomy 3, M3 = Monosomy 3, TG = Test Group, OG = Observational Group

A total of 186 patients participated in the present study. One hundred twelve patients formed the Observational Group, 74 opted for genetic testing and comprised the Test Group. Due to unclear biopsy results or dropout before t2 (no classification to M3 or D3 possible), 16 participants were excluded from the original Test Group. Detailed information about the number of patients in each group at each assessment point is provided in Fig. 2. The return rate of questionnaires per measurement point ranged from 78.3 (t1) – 100% (t0), dropouts already considered.

### Decision-making: characteristics of patients opting for genetic testing

Sample characteristics (see Table 2) are displayed for patients of the Test Group who participated at least until result announcement at t2 (Test Group Total: *n* = 63). Included are those who had an unclear biopsy result (*n* = 2) or were diagnosed with partial monosomy 3 (*n* = 3) in which case prognostication remains ambiguous [4, 5]. For patients who did not avail of testing (Observational Group: *n* = 112), all data will be depicted irrespective of drop-outs at any point of the study. To display significant differences between groups, unadjusted *p*-values are displayed for each characteristic. The correlation matrix can be viewed in the additional files (Additional file 1).

**Table 2:** Sociodemographic and psychosocial parameters of Test Group and Observational Group at t0

	Test Group Total* *n* = 63 (36%)	Observational Group *n* = 112 (64%)	Statistical results	*p*
Age (M, SD, range)	57.68 (±12.03), 29–82	63.08 (±11.4), 37–84	U = 2604.50, z = −2.872	.004** ^**a**^
Sex				.056^b^
male	42 (66.7%)	58 (51.8%)		
female	21 (33.3%)	54 (48.2%)		
Graduation				.196^b^
Intermediate school or less (< 12 years)	40 (63.5%)	82 (73.2%)		
High School or higher	22 (34.9%)	29 (25.9%)		
No information	1 (1.6%)	1 (0.9%)		
Treatment			χ^2^ = 34.666	<.001**^d^
Enucleation	23 (36.51%)	8 (7.14%)		
Brachytherapy	25 (39.68%)	90 (80.36%)		
Endoresection with adjuvant brachytherapy	7 (11.11%)	3 (2.68%)		
Proton therapy	6 (9.52%)	7 (6.25%)		
Other	2 (3.17%)	4 (3.57%)		
Resilience	70.08 (±11.16)	70.21 (±11.72)		.735^a^
Social Support	4.36 (±0.76)	4.51 (±0.54)		.499^a^
Fear of progression Total	2.34 (±0.72)	2.13 (±0.65)		.057^a^
Affective reactions	2.65 (±0.79)	2.57 (±0.73)		.601^c^
Partnership/Family	2.18 (±0.72)	2.02 (±0.66)		.134^a^
Occupation	2.23 (±1.18)	1.77 (±1.02)	U = 2738.0, z = −2.510	.012**^a^
Loss of autonomy	2.30 (±0.79)	2.17 (±0.85)		.202^a^
Coping with anxiety	3.46 (±0.55)	3.63 (±0.55)		.058^c^
General Distress	6.15 (±2.53)	5.40 (±2.44)	U = 2874.0, z = −2.047	.041**^a^
Depression	5.10 (±3.76)	4.90 (±4.30)		.498^a^
Anxiety	7.62 (±4.15)	7.21 (±3.87)		.519^c^
Health-related quality of life				
Quality of life: Physical	49.93 (±7.74)	48.22 (±9.65)		.463^a^
Quality of life: Mental	45.74 (±10.22)	44.57 (±11.82)		.682^a^
Perceived Risk	5.02 (±2.34)	4.06 (±2.31)	t(173) = 2.627	.009**^c^

Note: Except as indicated, categorical data are presented as count (percentage), continuous data are presented as mean (±standard deviations), * Test Group Total = M3 (*n* = 31) + D3 (*n* = 27) + unclear biopsy (*n* = 5), ** p < .05, a = Mann-Whitney U-Test, b = Chi-Square Test, c = Independent t-Test, d = Fisher’s exact test

Differences in sociodemographic parameters between those who opted for testing (and decided to receive the results = Test Group Total) and those who opted out (Observational Group) could only be found for age. Patients who opted for testing were significantly younger than those who did not avail of prognostication (*p* = .004). Treatment methods significantly differed between the two groups (p = <.001). We revealed group differences in psychosocial factors for general distress (*p* = .041) and perceived risk of metastases. Participants of the Test Group showed higher general distress and a greater risk of metastases than those in the Observational Group (*p* = .009). Fear of progression concerning one’s employment (occupation) was significantly higher in the Test Group (*p* = .012), whereas the total score only showed a significant trend (*p* = .057). The binary logistic regression model is depicted in Table 3. All sociodemographic, psychosocial parameters, and treatment methods were used as predictors. Treatment method (enucleated patients were more likely to opt for testing than those treated with brachytherapy), lower social support, and higher general distress could significantly predict the utilization of prognostic genetic testing. Perceived risk of metastases did not reach significance in the regression model (*p* = .293). Age only showed a trend towards significance (*p* = .079).

**Table 3:** Binary logistic regression model: Which patients opt for genetic testing?

Parameters	B (SE)	Wald	OR	95% CI	*p*
Age	−.035 (.020)	3.077	.966	.929–1.004	.079
Sex					
male	Ref.				
female	.658	2.175	1.931	.805–4.628	.140
Graduation					
Intermediate school or less (< 12 years)	Ref.				
High School or higher	.123 (.443)	.076	1.13	.474–2.695	.782
Resilience	.034 (.031)	1.186	1.034	.973–1.099	.276
Social Support	−.897 (.406)	4.889	.408	.184–.903	.027*
Fear of progression (Total)	.413 (.476)	.754	1.512	.595–3.841	.385
General Distress	.301 (.120)	6.272	1.351	1.068–1.710	.012*
Depression	.021 (.081)	.065	1.021	.871–1.196	.799
Anxiety	−.070 (.094)	.567	.932	.776–1-1.119	.451
Quality of Life: Physical	.044 (.028)	2.516	1.045	.990–1-103	.113
Quality of Life: Mental	.044 (.027)	2.679	1.045	.991–1.100	.102
Perceived Risk	.107 (.101)	1.106	1.112	.912–1.357	.293
Treatment		20.585			<.001**
Brachytherapy	Reference				
Enucleation	2.419 (.548)	19.513	11.233	3.841–32.853	<.001**
Proton therapy	.892 (.713)	1.565	2.441	.603–9.874	.211
Endoresection with adjuvant brachytherapy	1.249 (.876)	2.033	3.488	.626–19.421	.154

Note: R^2^ = .286 (Cox&Snell), .393 (Nagelkerke), −2Log-Likelihood = 157.962, Model χ^2^(15) = 55.133, **p < .001, * p < .05; Ref. = Reference, OR = Odds Ratio, CI = confidence interval

### Psychological distress and utilization of psycho-oncological interventions and its development after genetic testing

#### Psychological distress of genetic testing and its development over time

We examined whether psychosocial parameters changed after receiving the prognosis (t2) and differed from the Observational Group, while controlling for age, sex, and enucleation. All corresponding figures are available in the additional files (Additional file 2, Additional file 3, Additional file 4, Additional file 5, Additional file 6 and Additional file 7).

#### Perceived risk of metastases

**Fig. 3:**
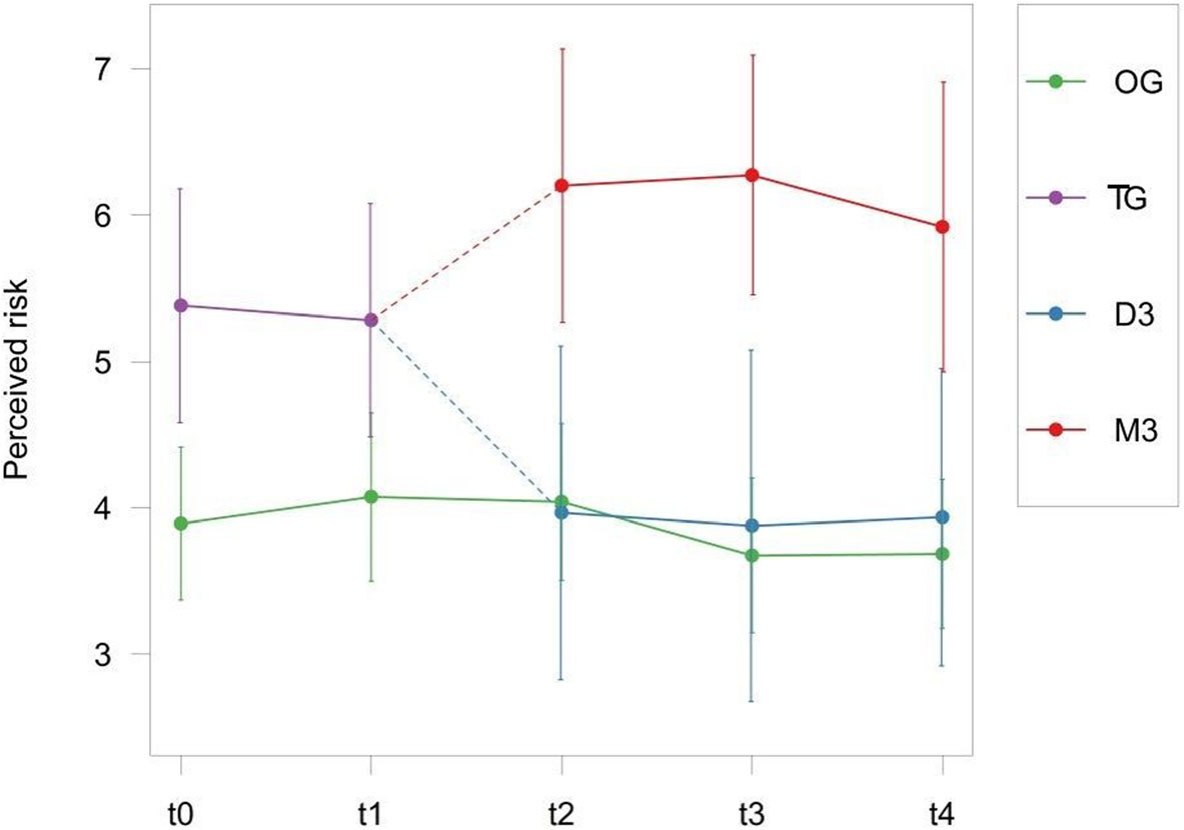
Unadjusted means of Perceived Risk over time per group. D3 = Disomy 3, M3 = Monosomy 3, TG = Test Group, OG = Observational Group

Figure 3 displays the course of perceived risk of metastases per group over time. Before prognostication, patients opting for genetic testing (Test Group) displayed significantly higher perceived risk of metastases than patients declining (Observational Group) it (estimated coefficient [SE], 1.06 [.35], *p* = .003). After announcement of test results, patients diagnosed with Monosomy 3 showed a significant increase in their perceived risk of metastases than before (.55 [.28], *p* = .048). In patients receiving the more favorable diagnosis of Disomy 3, a significant decrease appeared (−.91 [28], *p* = .001). After prognostication, patients with D3 did no longer differ from the Observational Group (.15 [.39], *p* = .695), whereas patients with M3 showed a distinctly higher level of perceived risk (1.61 [.39], *p* < .001). No overall time effect could be found (−.04 [.05], *p* = .39). The results are displayed in detail in Table 4.

**Table 4:** Mixed Model: Perceived Risk of Metastases

	Contrast: Before and after diagnosis			Contrast: Observational Group		
	Mean (SE)	t-value	*p*	Mean (SE)	t-value	*p*
Intercept	4.93 (.87)	5.69	<.001**	4.93 (.87)	5.69	<.001**
Test Group (before prognosis)	1.06 (.35)	3.00	.003**	1.06 (.35)	3.00	.003**
D3 (after prognosis)	−.91 (.28)	−3.23	.001**	.15 (.39)	.39	.695
M3 (after prognosis)	.55 (.28)	1.98	.048*	1.61 (.39)	4.12	<.001**
enucleation	.05 (.29)	.18	.855	.05 (.29)	.18	.855
time	−.04 (.05)	−.86	.391	−.04 (.05)	−.86	.391
age	−.01 (.01)	−.84	.404	−.01 (.01)	−.84	.404
sex	−.21 (.31)	−.67	.503	−.21 (.31)	−.67	.503

Note: In the first three columns (contrast: before and after diagnosis), the effects of D3 and M3 describe a change in perceived risk after prognosis when compared to before. In the last three columns (contrast: Observational Group), levels in perceived risk of D3 and M3 are compared to the Observational Group after prognosis; * p < .05; ** *p* < .005. *D3* Disomy 3, *M3* Monosomy 3

#### Fear of progression

Fear of progression significantly decreased both in patients with M3 (−.11 [.05], *p* = .02) and D3 (−.11 [.05], p = .003) after receiving the prognosis. Neither patients with D3 (−.03 [.11], *p* = .773) nor M3 (.01 [.11], *p* = .95) differed significantly from the Observational Group after diagnosis. We also found an overall time effect indicating a decrease in fear in all groups (−.04 [.01], p < .001).

#### General distress

Result announcement was not associated with a significant change in general distress, neither for patients with the good prognosis (D3) (.17 [.32], *p* = .60), nor for those with the poor one (M3) (−.08 [.31], *p* = .80). No difference in general distress was found for M3 (.37 [.40], *p* = .35) nor for D3 (.63, [.40], *p* = .12) compared to the Observational Group after prognostication. A decrease of general distress over time was found (−.31 [.05], *p* < .001).

#### Depression

Depressive symptoms significantly decreased after receiving prognostication results both in patients with M3 (−.95 [.39], *p* = .01] and D3 (−.84 [.39], *p* = .03). Moreover, there was a significant declining time effect of depression over the course of the study in all groups (−.13 [.06], *p* = .04). After prognostication, depression scores of patients with M3 (−.34 [.71], *p* = .64) and D3 (−.22 [.71], *p* = .75) did not differ from depression scores in the Observational Group. The mean scores of all patients stayed below the cut-off score for clinically relevant depression at each time point.

#### Anxiety

Right after receiving the prognosis, anxiety scores revealed a declining trend in both groups (M3 and D3), but neither could reach significance (M3: −.55 [.39], *p* = .16; D3: −.76 [.40], *p* = .056). However, a significant decrease of anxiety could be observed in all groups continuously over time (−.34 [.06], *p* < .001). Neither M3 (.09 [.65], *p* = .88) nor D3 (−.12 [.65], *p* = .86) differed significantly from the Observational Group in their level of anxiety after receiving the results. The mean scores of all patients did not exceed the cut-off for pathological anxiety at any point of time.

#### Quality of life: mental

There was no change in mental quality of life after result announcement for both patients with M3 (.10 [1.27], *p* = .93) and D3 (.06 [1.28], *p* = .96). In neither group (M3: −.01 [1.79], *p* = .99; D3: −.05 [1.76], *p* = .98) did mental quality of life differ from the Observational Group after prognosis. However, there was a significant increase of mental quality of life during the course of the study in all groups (.89 [.21], *p* < .001).

#### Quality of life: physical

After diagnosis, physical quality of life did not change significantly for patients with M3 (1.64 [1.16], p = .16), nor for D3 (−.23 [1.17], *p* = .84). When compared to the Observational Group, patients receiving the poor diagnosis (M3) showed a significantly higher physical quality of life when compared to the mean score of the Observational Group (3.72 [1.61], *p* = .02), whereas patients with D3 did not differ significantly from the Observational Group (1.84 [1.59], *p* = .25). While this initially seems to disagree with the pattern found in the unadjusted data (Additional file 7), the model further shows a significant reduction in physical quality of life attributed to enucleation (− 2.66 [1.21], *p* = .03), which was considerably more prevalent for patients with M3 (D3: 4/17, M3: 17/31). Moreover, random intercept estimates for patients with M3 were slightly lower on average compared to D3. Overall, there was no significant change over time (−.18 [.19], *p* = .35).

### Utilization of psycho-oncological interventions

The frequencies of utilization of psycho-oncological interventions are displayed in Table 5. After prognostication, utilization increased to a steady > 10% for each group. This increase over time was significant (−.33 [.06], p < .001) while controlling for enucleation and sex (age was excluded from the analyses due to failing convergence of the optimization algorithm). Prognostication, however, was not associated with a change in utilization, independent of diagnosis (M3: −.55 [.39], *p* = .16; D3: −.76 [.40], *p* = .06). Likewise, neither M3 patients (.09 [.65], *p* = .88) nor D3 patients (−.12 [.64], *p* = .86) differed significantly in the utilization of interventions after testing when compared to the Observational Group. We used no imputations to replace missing data, since information on the utilization of psychological services can be viewed as a sensitive topic and might be left out intentionally [40]. A binary logistic regression was conducted to investigate the characteristics of patients who stated to have utilized psycho-oncological interventions at least once during study course. As can be viewed in Table 6, female sex, higher general distress, and higher anxiety could significantly predict the utilization of psycho-oncological services at least once. Most patients reported very high satisfaction with the psycho-oncological service.

**Table 5:** Frequencies of utilization of psycho-oncological services during study period

	OG	D3	M3	Total
t0				
Yes (%)	1.8	0	6.5	2.4
No (%)	84.8	96.3	80.6	85.9
No information (%)	13.4	3.7	12.9	11.8
t1				
Yes (%)	3.5	8.7	8	5.3
No (%)	92.9	91.3	92	92.5
No information (%)	3.5	0	0	2.3
t2				
Yes (%)	10.5	16.7	10	11.4
No (%)	81,4	83.3	90	83.6
No information (%)	8.1	0	0	5
t3				
Yes (%)	10.6	16.7	13.3	12.2
No (%)	87.1	83.3	83.3	85.6
No information (%)	2.4	0	3,3	2.2
t4				
Yes (%)	12.3	14.8	26.7	15.5
No (%)	87.7	85.2	70	81
No information (%)	4.7	0	3.3	3.5

Note: *OG* Observational Group, *D3* Disomy 3, *M3* Monosomy 3

**Table 6:** Binary logistic regression model: Which patients utilize psycho-oncological interventions?

Parameters	B (SE)	Wald	OR	95% CI	*p*
Age	−.043 (.025)	2.869	.958	.985–1.007	.090
Sex					
male	Ref.				
female	−1.399 (.535)	6.838	.247	.087–.704	.009*
Graduation					
Intermediate school or less (< 12 years)	Ref.				
High School or higher	.592 (.553)	1.145	1.807	.611–5.342	.285
Resilience	−.059 (.034)	3.008	.943	.882–1.008	.083
Social Support	.252 (.515)	.239	1.287	.469–3.532	.625
Fear of progression (Total)	.536 (.489)	1.202	1.709	.656–4.455	.273
General Distress	.402 (.151)	7.108	1.494	1.112–2.007	.008*
Depression	.028 (.100)	.081	1.029	.846–1.250	.776
Anxiety	−.383 (.125)	9.346	.682	.533–.872	.002**
Quality of Life: Physical	.019 (.029)	.421	1.019	.963–1.078	.516
Quality of Life: Mental	−.003 (.029)	.013	.997	.941–1.055	.908
Perceived Risk	.149 (.124)	1.440	1.160	.910–1.479	.230
Enucleation	.414 (.634)	.427	1.513	.437–5.243	.513
No	Ref.				
Yes	.414 (.634)	.427	1.513	.437–5.243	.513
Group	.269 (.318)	.318	1.308	.701–2.440	.398
OG	Ref.	.717			.699
D3	.245 (.671)	.134	1.278	.343–4.764	.715
M3	.543 (.650)	.697	1.721	.481–6.152	.404

Note: R^2^ = .227 (Cox&Snell), .365 (Nagelkerke). Model χ^2^(14) = 41.97, * *p* < .05, **p < .005. *OG* Observational Group, *D3* Disomy 3, *M3* Monosomy 3

### Risk: which factors predict prolonged psychological distress?

Multiple linear regressions of patients’ characteristics were calculated to predict future psychological outcome (Depression, Anxiety, General Distress at t4). As can be seen in Table 7, a higher depressive score at the end of the study (t4) could significantly be predicted by lower resilience (*p* = .023), higher fear of progression (*p* = .010), lower physical quality of life (*p* = .022), and higher depression (*p* < .001) at the beginning of the study. A higher future anxiety score (t4) was significantly predicted by younger age (*p* = .031), higher fear of progression (*p* = .001), higher anxiety (*p* = .039), lower physical (*p* = .042), and mental (*p* = .043) quality of life at t0. Higher future General Distress (t4) could only be predicted by a higher level of General Distress (p < .001) at the beginning of the study (t0). Neither the mean score for anxiety (x¯ = 5.38, SE = 3.84) nor for depression (x¯ = 4.14, SE = 3.85) reached the cut-off for a clinically relevant condition at t4.

**Table 7:** Multiple linear regression: Which factors predict prolonged psychological distress?

	Depression Score t4^a^			Anxiety Score t4^b^			General Distress t4^c^		
	B (SE)	β	p	B (SE)	β	p	B (SE)	β	p
Constant	14.568 (3.761)		<.001*	9.464 (3.876)		.016*	4.874 (2.988)		.105
Age	−.021 (.022)	−.062	.342	−.050 (.023)	−.147	.031*	−.013 (.018)	−.059	.461
Sex	.711 (.482)	.090	.142	−.134 (.496)	−.017	.787	−.101 (.384)	−.020	.792
Graduation	.184 (.469)	.022	.696	.102 (.483)	.012	.834	.447 (.374)	.082	.235
Resilience	−.074 (.032)	−.220	.023*	−.011 (.033)	−.034	.735	−.022 (.026)	−.101	.404
Social Support	−.476 (.409)	−.079	.247	.130 (.421)	.022	.758	−.560 (.326)	−.143	.089
Fear of progression (Total)	1.326 (.508)	.224	.010*	1.744 (.524)	.298	.001*	.697 (.697)	.188	.082
General Distress	.085 (.132)	.055	.519	−.016 (.136)	−.011	.904	.452 (.105)	.450	<.001**
Depression	.418 (.090)	.432	<.001**	.112 (.092)	.117	.227	.082 (.072)	.132	.255
Anxiety	−.186 (.099)	−.186	.062	.212 (.102)	.215	.039*	−.077 (.077)	−.119	.322
Quality of Life: Physical	−.067 (.029)	−.142	.022*	−.061 (.030)	−.131	.042*	−.006 (.022)	−.021	.781
Quality of Life: Mental	−.044 (.029)	−.127	.131	−.061 (.030)	−.178	.043*	.022 (.023)	.099	.341
Perceived Risk	−.153 (.113)	−.093	.179	−.066 (.116)	−.041	.572	−.003 (.090)	−.003	.971
Enucleation	.998 (.637)	.097	.119	.692 (.656)	.068	.293	.054 (.509)	.008	.915
Group (M3, D3, OG)	−.296 (.313)	−.062	.346	−.362 (.323)	−.076	.264	−.226 (.250)	−.073	.368

Note: * *p* < .05; ** *p* < .001; ^a^ R^2^ = .643, F (14) = 15.952, *p* < .001, ^b^R^2^ = .612, F(14) = 13.993, *p* < .001; ^c^R^2^ = .453, F(14) = 7.4, *p* < .001. *OG* Observational Group, Disomy 3, *M3* Monosomy 3

## Discussion

This was a non-randomized controlled prospective trial on the psychosocial impact of prognostic genetic testing, where participants were assessed at 5 different time points, before and after testing.

### Decision-making: characteristics of patients opting for genetic testing

A total of 63 patients (36%) decided to receive the results on their prognosis which is in line with the study of Beran and colleagues [9]. We examined several factors contributing to this choice and found that treatment method (enucleation > brachytherapy), lower social support, and higher general distress could significantly predict the utilization of prognostic genetic testing. Treatment methods seem to play an important role in decision-making. Especially enucleated patients are more likely to opt for prognostication than those treated with brachytherapy. The potentially higher burden associated with this treatment [15, 17, 18] could be associated with the need of more intensive care and support, hoped to be met by study participation. Additionally, enucleation provides the possibility of sample removal from the enucleated eye right after surgery and thus could facilitate the decision for prognostication. Patients treated with other methods (e.g. brachytherapy, proton therapy) face the obstacle of additional surgery for biopsy if they wish to receive information on their prognosis. Although, no difference in treatment could be found by Beran and colleagues [9], plaque radiotherapy was the main treatment used, while only few patients were enucleated. This research group [9] further found male sex to be significantly associated with the decision for testing. Equal results could be attained in our study, although no significance could be reached. Moreover, we found higher general distress and lower social support to be predictive for the use of genetic testing. Considering that highly distressed patients with low social support opt for testing, especially in situations where rational judgement is problematic [24] and shared decisions are preferred [25], professional support (e.g. an decision aid intervention [41]) during the decision-making process seems indispensable.

### Psychological distress and utilization of psycho-oncological interventions and its development after genetic testing

We found that depression, anxiety, general distress, and fear of progression declined while mental quality of life increased continuously over time independent of diagnosis (M3 or D3) or group allocation (Test Group or Observational Group). This is in line with previous research that found similar time effects for anxiety [11], depression [9, 11], and quality of life [9] in patients with uveal melanoma. This also fits the overall assumption that genetic testing does not affect the psychological status of UM patients in a negative way [8, 10, 42]. In our study, genetic testing was associated with an immediate reduction in fear of progression and depressive symptoms, irrespective of whether patients were informed they had a good or a poor prognosis. While perceived risk of metastases increased following prognostication in those with a poor prognosis, it declined to the same level as in the observation group among those with a good prognosis. Physical quality of life did not change after result announcement; it was significantly increased for patients with M3, though, when compared to the Observational Group. Prognostication in UM seems to be associated with aspects of psychosocial well-being in different ways. The increased level of perceived risk of metastases in our high risk group (M3) and a decreased level in the low risk group (D3) after genetic testing correspond to the actual medical risk and suggests an improved accuracy of risk perception similar to hereditary testing in other cancer types [43–45]. The immediate decline of depression and fear of progression after prognostication might be the result of relief or acceptance by gaining certainty after a certain waiting period. As presumed in previous studies [8, 24], M3 patients receiving the high risk prognosis might also consider it as hopeful and associate it with more follow-up visits increasing the chance of metastases detection despite low survival chances. Physical Quality of Life did not change significantly after prognostication, but was significantly increased in M3 compared to the Observational Group when adjusting for enucleation and unobserved variability in the linear mixed model (see Result section). This leads to the conclusion that enucleation might impact patients severely in their physical quality of life, whereas the information on their genetic status seems to be of less importance. This is in line with other studies, where physical quality of life was found to be independent of prognosis [8, 12].

Overall, our results could also be explained by the increasing frequency of utilization of psycho-oncological interventions we found over the course of the study. While a growing number of patients took advantage of psychological support, psychological distress decreased either right after prognosis or over time. Yet, the perceived risk of metastases was assessed adequately. Taking into account the overall satisfaction with the psycho-oncological service during the study, psychological support seems to be an essential component, before, during, and after prognostic testing and should be routinely implemented in this process [9, 10, 27]. Especially highly distressed, anxious female patients seem to form a prominent target group, that is more likely to seek support.

What else needs to be mentioned is that some studies found continuously increased depressive scores and distress as well as lower quality of life in patients with malignant uveal melanoma after treatment [12, 13]. Research still lacks clarity on whether different treatment methods impact psychosocial well-being [10, 12, 14–21]. This is important, especially if specific treatments can be interrelated with the nature of the tumor or a certain diagnosis [7, 46, 47]. In our study, we controlled for enucleation to adjust for potential impairments associated with this treatment (loss of eye and sight, cosmetic changes etc. [17, 18], and found a significant association with Physical Quality of Life. Therefore, it is crucial for future studies on psychosocial impact to distinguish more between the potential impact of treatment methods and the impact of prognostication, in order to gain more insight and receive even more specific results.

### Risk: which factors predict prolonged psychological distress?

In our study, lower resilience, higher fear of progression, lower physical quality of life, and a higher depression score at the beginning of the study significantly predicted a higher depression score at the end of the study. Future anxiety as well as future distress could equally be predicted by their respective baseline levels. The respective groups (M3, D3, Observational Group) had no significant influence on any psychological outcome. No studies on factors predicting prolonged psychological distress in UM patients could be identified. We chose to relate to literature on other types of cancer, since we assume similarities in the psychological adjustment of cancer patients over time. A study on hereditary breast cancer patients showed that baseline anxiety and depressive scores could also significantly predict depression and anxiety after 5-8 years [48] while another study found physical function (similar to physical quality of life in our study) to be associated with better psychological adjustment after breast cancer [49]. This is also in line with our findings on anxiety which revealed that anxiety at the end of the study could be predicted by physical and mental quality of life. We further found fear of progression and younger age to be predictive for future anxiety. The latter is congruous with a study suggesting that younger (hematologic) cancer patients have the highest risk to experience both depression and anxiety [50]. However, it has to be taken into account that the mean score of both depression and anxiety did not exceed the cut-off for a pathological condition at any point in our study.

### Summary, limitations and future prospect

To the best of our knowledge, this is the first controlled prospective study on the psychosocial impact of patients diagnosed with uveal melanoma entailing major implications for clinical practice and patient care. Overall, we found that genetic testing is not associated with poorer subsequent psychological well-being. It rather reduces adverse mental conditions and improves the accuracy of risk perception. However, psychological support should be available to all patients, independent of prognosis and treatment. Special attention should be paid to highly distressed, anxious, and female patients as a group with increased utilization of interventions. In ophthalmologic clinics, risk factors for psychological distress (e.g. depression, anxiety, fear of progression, low quality of life, general distress, and young age) should be routinely screened for with adequate instruments [51], to provide appropriate and immediate psychological support if necessary.

One of the major strengths of this study was the inclusion of an observational group not opting for testing and the investigation of a large variety of psychosocial factors compared to previous studies [10]. However, randomization and blinding was not feasible. Due to the relatively small sample size of patients opting for prognostication, conclusions and generalization should be made with caution. Further studies with greater samples are recommended.

What is more, treatment methods such as enucleation might impact certain psychological variables far more than genetic testing. Therefore, future research should consider the treatment method as a potential factor of distress when investigating the impact of genetic testing, in order to be able to examine their effects separately. Studies should further examine how interventions can influence the coping with prognostic testing and in which way those interventions could be improved and tailored to the specific needs of patients with UM [26]. The examination of trait measures like risk propensity, health literacy, and numeracy could provide more information on how the choice is made by patients and how they could mediate the emotional impact of the results report. Also further qualitative research comparable to Hope-Stone and colleagues [23] is advisable in order to shed more light on different aspects of psychosocial well-being. Future research would also benefit from investigating this topic in different contexts (eg. patients’ comorbidities, different clinical history, other diseases), which could provide an interesting continuation of our study.

## Supplementary information

**Additional file 1.** Correlation matrix.

**Additional file 2.** Unadjusted means of Fear of Progression over time per group. OG = Observational Group, TG = Test Group, D3 = Disomy 3, M3 = Monosomy 3.

**Additional file 3.** Unadjusted means of General Distress over time per group. OG = Observational Group, TG = Test Group, D3 = Disomy 3, M3 = Monosomy 3.

**Additional file 4.** Unadjusted means of Depression over time per group. OG = Observational Group, TG = Test Group, D3 = Disomy 3, M3 = Monosomy 3.

**Additional file 5.** Unadjusted means of Anxiety over time per group. OG = Observational Group, TG = Test Group, D3 = Disomy 3, M3 = Monosomy 3.

**Additional file 6.** Unadjusted means of Mental Quality of Life over time per group. OG = Observational Group, TG = Test Group, D3 = Disomy 3, M3 = Monosomy 3.

**Additional file 7.** Unadjusted means of Physical Quality of Life over time per group. OG = Observational Group, TG = Test Group, D3 = Disomy 3, M3 = Monosomy 3.

## Data Availability

The data supporting our findings can be requested from Dipl.-Psych. Marietta Lieb (marietta.lieb@uk-erlangen.de) and Prof. Yesim Erim (yesim.erim@uk-erlangen.de).

## Abbreviations

CI: Confidence Interval
D3: Disomy 3
M3: Monosomy 3
OG: Observational Group
OR: Odds Ratio
Ref.: Reference
TG: Test Group
VAS: Visual Analogue Scale

## References

[1] Virgili G, Gatta G, Ciccolallo L, Capocaccia R, Biggeri A, Crocetti E (2007). Incidence of uveal melanoma in Europe. Ophthalmology..

[2] Schopper VJ, Correa ZM (2016). Clinical application of genetic testing for posterior uveal melanoma. Int J Retina Vitreous.

[3] Prescher G, Bornfeld N, Hirche H, Horsthemke B, Jockel KH, Becher R (1996). Prognostic implications of monosomy 3 in uveal melanoma. Lancet..

[4] Damato B, Dopierala JA, Coupland SE (2010). Genotypic profiling of 452 choroidal melanomas with multiplex ligation-dependent probe amplification. Clin Cancer Res.

[5] Thomas S, Putter C, Weber S, Bornfeld N, Lohmann DR, Zeschnigk M (2012). Prognostic significance of chromosome 3 alterations determined by microsatellite analysis in uveal melanoma: a long-term follow-up study. Br J Cancer.

[6] Metz CH, Lohmann D, Zeschnigk M, Bornfeld N (2013). Uveal melanoma: current insights into clinical relevance of genetic testing. Klin Monatsbl Augenheilkd.

[7] Dogrusoz M, Jager MJ (2018). Genetic prognostication in uveal melanoma. Acta Ophthalmol.

[8] Cook SA, Damato B, Marshall E, Salmon P (2009). Psychological aspects of cytogenetic testing of uveal melanoma: preliminary findings and directions for future research. Eye (Lond)..

[9] Beran TM, McCannel TA, Stanton AL, Straatsma BR, Burgess BL (2009). Reactions to and desire for prognostic testing in choroidal melanoma patients. J Genet Couns.

[10] Miniati M, Fabrini MG, Genovesi Ebert F, Mancino M, Maglio A, Massimetti G (2018). Quality of life, depression, and anxiety in patients with Uveal melanoma: a review. J Oncol.

[11] Schuermeyer I, Maican A, Sharp R, Bena J, Triozzi PL, Singh AD (2016). Depression, anxiety, and regret before and after testing to estimate Uveal melanoma prognosis. JAMA Ophthalmol.

[12] Hope-Stone L, Brown SL, Heimann H, Damato B, Salmon P (2016). Two-year patient-reported outcomes following treatment of uveal melanoma. Eye (Lond).

[13] Reimer J, Voigtlaender-Fleiss A, Karow A, Bornfeld N, Esser J, Helga FG (2006). The impact of diagnosis and plaque radiotherapy treatment of malignant choroidal melanoma on patients’ quality of life. Psychooncology..

[14] Amaro TA, Yazigi L, Erwenne C (2010). Depression and quality of life during treatment of ocular bulb removal in individuals with uveal melanoma. Eur J Cancer Care (Engl).

[15] Wiley JF, Laird K, Beran T, McCannel TA, Stanton AL (2013). Quality of life and cancer-related needs in patients with choroidal melanoma. Br J Ophthalmol.

[16] Melia M, Moy CS, Reynolds SM, Hayman JA, Murray TG, Hovland KR (2006). Quality of life after iodine 125 brachytherapy vs enucleation for choroidal melanoma: 5-year results from the collaborative ocular melanoma study: COMS QOLS report no. 3. Arch Ophthalmol.

[17] Frenkel S, Rosenne H, Briscoe D, Hendler K, Bereket R, Molcho M (2018). Long-term uveal melanoma survivors: measuring their quality of life. Acta Ophthalmol.

[18] Klingenstein A, Furweger C, Nentwich MM, Schaller UC, Foerster PI, Wowra B (2013). Quality of life in the follow-up of uveal melanoma patients after CyberKnife treatment. Melanoma Res.

[19] Blanco-Rivera C, Capeans-Tome C, Otero-Cepeda XL (2008). Quality of life in patients with choroidal melanoma. Arch Soc Esp Oftalmol.

[20] Brandberg Y, Kock E, Oskar K, af Trampe E, Seregard S (2000). Psychological reactions and quality of life in patients with posterior uveal melanoma treated with ruthenium plaque therapy or enucleation: a one year follow-up study. Eye (Lond)..

[21] Chabert S, Velikay-Parel M, Zehetmayer M (2004). Influence of uveal melanoma therapy on patients’ quality of life: a psychological study. Acta Ophthalmol Scand.

[22] Vos J, Gomez-Garcia E, Oosterwijk JC, Menko FH, Stoel RD, van Asperen CJ (2012). Opening the psychological black box in genetic counseling. The psychological impact of DNA testing is predicted by the counselees’ perception, the medical impact by the pathogenic or uninformative BRCA1/2-result. Psychooncology..

[23] Hope-Stone L, Brown SL, Heimann H, Damato B, Salmon P (2015). How do patients with uveal melanoma experience and manage uncertainty? A qualitative study. Psychooncology..

[24] Cook SA, Damato B, Marshall E, Salmon P (2011). Reconciling the principle of patient autonomy with the practice of informed consent: decision-making about prognostication in uveal melanoma. Health Expect.

[25] Deber RB, Kraetschmer N, Urowitz S, Sharpe N (2007). Do people want to be autonomous patients? Preferred roles in treatment decision-making in several patient populations. Health Expect.

[26] Williamson TJ, Jorge-Miller A, McCannel TA, Beran TM, Stanton AL (2018). Sociodemographic, medical, and psychosocial factors associated with supportive care needs in adults diagnosed with Uveal melanoma. JAMA Ophthalmol..

[27] Phelps C, Bennett P, Hood K, Brain K, Murray A (2013). A self-help coping intervention can reduce anxiety and avoidant health behaviours whilst waiting for cancer genetic risk information: results of a phase III randomised trial. Psychooncology..

[28] Antonovsky A (1993). The structure and properties of the sense of coherence scale. Soc Sci Med.

[29] Fydrich T, Sommer G, Menzel U, Höll B (1987). Fragebogen zur sozialen Unterstützung (Kurzform; SozU-K-22). Z Klin Psychol.

[30] Sommer G, Fydrich T. Soziale Unterstützung: Diagnostik, Konzepte, F-SozU. Tübingen: Dt. Ges. für Verhaltenstherapie; 1989.

[31] Sommer G, Fydrich T (1991). Entwicklung und Überprüfung eines Fragebogens zur sozialen Unterstützung. Diagnostica..

[32] Herschbach P, Berg P, Dankert A, Duran G, Engst-Hastreiter U, Waadt S (2005). Fear of progression in chronic diseases: psychometric properties of the fear of progression questionnaire. J Psychosom Res.

[33] Herschbach P, Dankert A, Duran-Atzinger G, Waadt S, Engst-Hastreiter U, Keller M (2001). Diagnostik von Progredienzangst – Entwicklung eines Fragebogens zur Erfassung von Progredienzangst bei Patienten mit Krebserkrankungen, Diabetes mellitus und entzündlich-rheumatischen Erkrankungen in der Rehabilitation.

[34] Roth AJ, Kornblith AB, Batel-Copel L, Peabody E, Scher HI, Holland JC (1998). Rapid screening for psychologic distress in men with prostate carcinoma: a pilot study. Cancer..

[35] Herrmann C, Buss U, Snaith RP (1995). HADS-D - hospital anxiety and depression scale - deutsche version.

[36] Ware J, Kosinski M, Keller SD (1996). A 12-item short-form health survey: construction of scales and preliminary tests of reliability and validity. Med Care.

[37] Pinheiro J, Bates D, DebRoy S, Sarkar D, Team RC (2014). Nlme: linear and nonlinear mixed effect models. R Package Version.

[38] Bates D, Mächler M, \ker B, Walker S (2015). Fitting linear mixed-effects models using lme4. J Stat Softw.

[39] R.Core.Team (2018). R: A language and environment for statistical computing.

[40] Reddy MK, Fleming MT, Howells NL, Rabenhorst MM, Casselman R, Rosenbaum A (2006). Effects of method on participants and disclosure rates in research on sensitive topics. Violence Vict.

[41] Wakefield CE, Meiser B, Homewood J, Ward R, O'Donnell S, Kirk J (2008). Randomized trial of a decision aid for individuals considering genetic testing for hereditary nonpolyposis colorectal cancer risk. Cancer..

[42] Meiser B (2005). Psychological impact of genetic testing for cancer susceptibility: an update of the literature. Psychooncology..

[43] Braithwaite D, Emery J, Walter F, Prevost AT, Sutton S (2006). Psychological impact of genetic counseling for familial cancer: a systematic review and meta-analysis. Familial Cancer.

[44] Shiloh S, Dagan E, Friedman I, Blank N, Friedman E (2013). A follow-up study on men tested for BRCA1/BRCA2 mutations: impacts and coping processes. Psychooncology..

[45] Meiser B, Halliday JL (2002). What is the impact of genetic counselling in women at increased risk of developing hereditary breast cancer? A meta-analytic review. Soc Sci Med.

[46] Shields CL, Say EAT, Hasanreisoglu M, Saktanasate J, Lawson BM, Landy JE (2017). Cytogenetic abnormalities in Uveal melanoma based on tumor features and size in 1059 patients: the 2016 W. Richard Green Lecture. Ophthalmology.

[47] Le Guin CHD, Metz KA, Lehmann N, Kreis SH, Bornfeld N, Rudolf Lohmann D (2019). Chromosome 3 is a valid marker for prognostic testing of biopsy material from uveal melanoma later treated by brachytherapy. Biomarkers..

[48] den Heijer M, Seynaeve C, Vanheusden K, Timman R, Duivenvoorden HJ, Tilanus-Linthorst M (2013). Long-term psychological distress in women at risk for hereditary breast cancer adhering to regular surveillance: a risk profile. Psychooncology..

[49] Brandao T, Schulz MS, Matos PM (2017). Psychological adjustment after breast cancer: a systematic review of longitudinal studies. Psychooncology..

[50] Kuba K, Esser P, Mehnert A, Hinz A, Johansen C, Lordick F (2019). Risk for depression and anxiety in long-term survivors of hematologic cancer. Health Psychol.

[51] Erim Y, Beckmann M, Gerlach G, Kümmel S, Oberhoff C, Senf W (2009). Screening von psychischen Belastungen bei erst erkrankten Brustkrebspatientinnen: Einsatz von HADS-D und PO-Bado [screening for distress in women with breast cancer diagnosed for the first time: employment of HADS-D and PO-Bado]. Z Psychosom Med Psychother.

