# Supplementary material for: Psychosocial impact of prognostic genetic testing in uveal melanoma patients: a controlled prospective clinical observational study
Source: BMC Psychol. 2020 Jan 31;8:8. doi: 10.1186/s40359-020-0371-3 (PMC6995105; doi:10.1186/s40359-020-0371-3)
Supplement: Supplementary file 1 — Additional file 1. Correlation matrix. [file 40359_2020_371_MOESM1_ESM.pdf]

	**Age**	**Sex**	**Resilience**	**Social Support**	**Fear of Progression**	**General Distress**	**Depression**	**Anxiety**	**Quality of Life: Physical**	**Quality of Life: Mental**	**Perceived Risk**
**Age**	xx	**.021***	.128	-.138	**-.395****	**-.292****	-.006	**-.207****	**-.176***	**.212****	-.099
**Sex**	**.021***	xx	**297****	-.067	**-.179***	-.075	**-.206****	**-.288****	.043	258**	-.070
**Resilience**	.128	**.297****	xx	**.164****	**-.582****	**-.514****	**-.694****	**-.616****	**.339****	**.573****	**-.415****
**Social Support**	-.138	-.067	**.164****	xx	**-.152***	-.107	**-.409****	-.090	**.241****	.141	**-.174***
**Fear of Progression**	**-.395****	**-.179***	**-.582****	**-.152***	xx	**.625****	**.575****	**.692****	**-.170***	**-.552****	**.342****
**General Distress**	**-.292****	-.075	**-.514****	-.107	**.625****	xx	**.496****	**.580****	**-.232****	**-.538****	**.297****
**Depression**	-.006	**-.206****	**-.694****	**-.409****	**.575****	**.496****	xx	**.656****	**-.321****	**-.597****	**.387****
**Anxiety**	**-.207****	**-.288****	**-.616****	-.090	**.692****	**.580****	**.656****	xx	**-.155***	**-.618****	**.413****
**Quality of Life: Physical**	**-.176***	.043	**.339****	**.241****	**-.170***	**-.232****	**-.321****	**-.155***	xx	.106	-.126
**Quality of Life: Mental**	**.212****	**.258****	**.573****	.141	**-.552****	**-.538****	**-.597****	**-.618****	.106	xx	**-.298****
**Perceived Risk**	-.099	-.070	**-.415****	**-.174***	**.342****	**.297****	**.387****	**.413****	-.126	**-.298****	xx

* $p < .05$, ** $p < .01$
