# Supplementary figures and images for: Psychosocial impact of prognostic genetic testing in uveal melanoma patients: a controlled prospective clinical observational study
Source: BMC Psychol. 2020 Jan 31;8:8. doi: 10.1186/s40359-020-0371-3 (PMC6995105; doi:10.1186/s40359-020-0371-3)

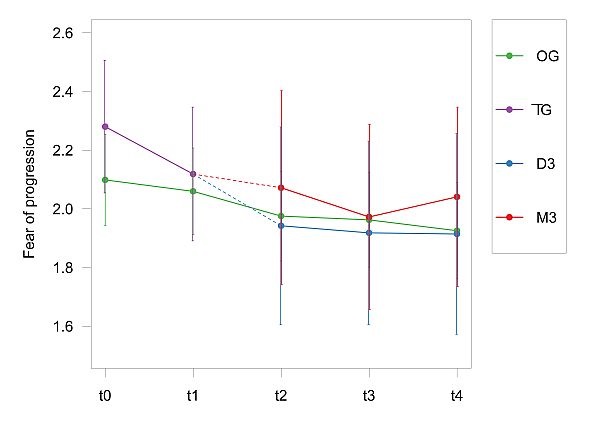

Supplement: Supplementary file 2 — Additional file 2. Unadjusted means of Fear of Progression over time per group. OG = Observational Group, TG = Test Group, D3 = Disomy 3, M3 = Monosomy 3. [file 40359_2020_371_MOESM2_ESM.jpg]

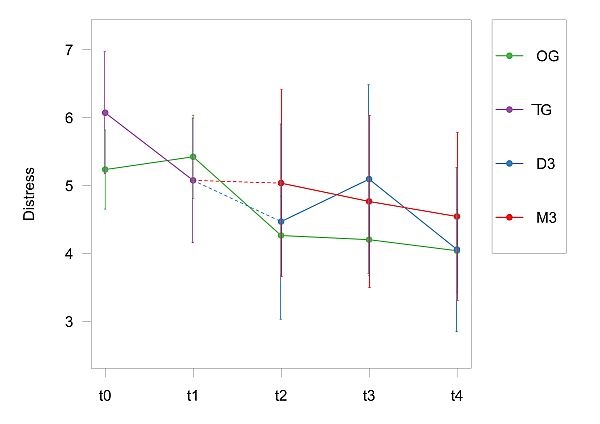

Supplement: Supplementary file 3 — Additional file 3. Unadjusted means of General Distress over time per group. OG = Observational Group, TG = Test Group, D3 = Disomy 3, M3 = Monosomy 3. [file 40359_2020_371_MOESM3_ESM.jpg]

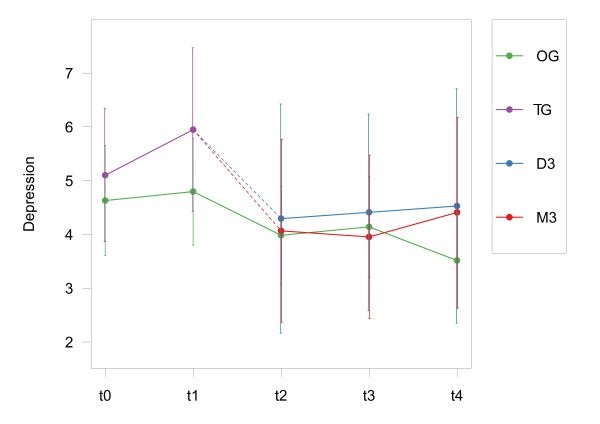

Supplement: Supplementary file 4 — Additional file 4. Unadjusted means of Depression over time per group. OG = Observational Group, TG = Test Group, D3 = Disomy 3, M3 = Monosomy 3. [file 40359_2020_371_MOESM4_ESM.jpg]

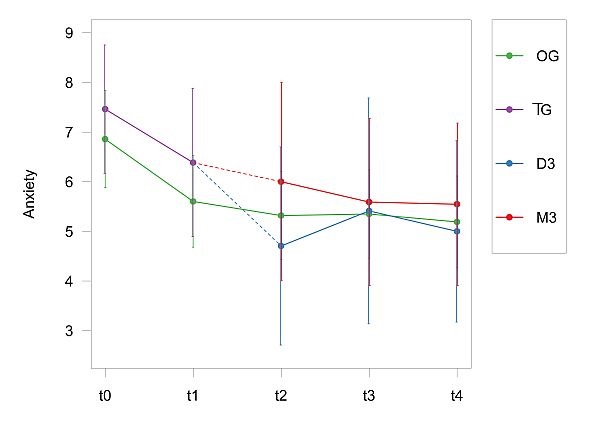

Supplement: Supplementary file 5 — Additional file 5. Unadjusted means of Anxiety over time per group. OG = Observational Group, TG = Test Group, D3 = Disomy 3, M3 = Monosomy 3. [file 40359_2020_371_MOESM5_ESM.jpg]

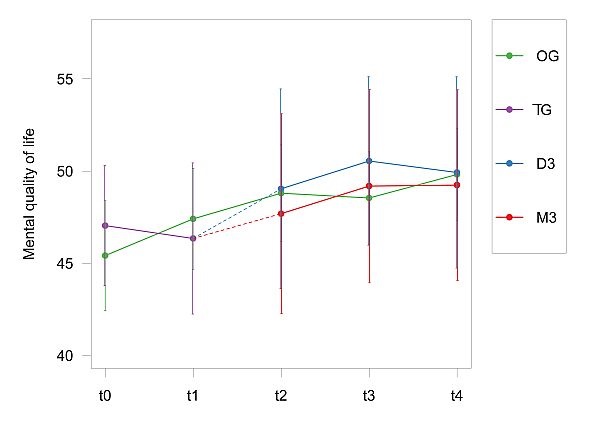

Supplement: Supplementary file 6 — Additional file 6. Unadjusted means of Mental Quality of Life over time per group. OG = Observational Group, TG = Test Group, D3 = Disomy 3, M3 = Monosomy 3. [file 40359_2020_371_MOESM6_ESM.jpg]

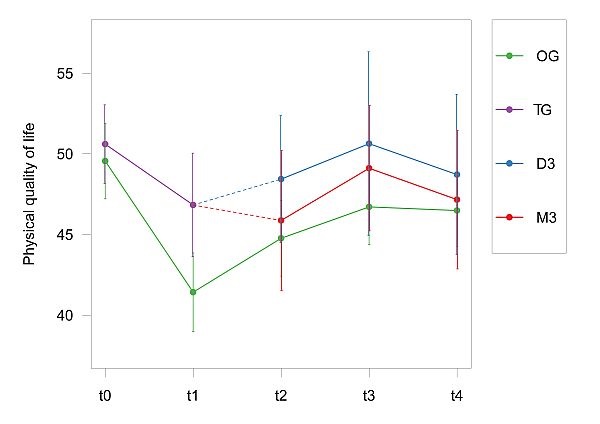

Supplement: Supplementary file 7 — Additional file 7. Unadjusted means of Physical Quality of Life over time per group. OG = Observational Group, TG = Test Group, D3 = Disomy 3, M3 = Monosomy 3. [file 40359_2020_371_MOESM7_ESM.jpg]
